# New Enhancement beyond Radiation Field Improves Survival Prediction in Patients with Post-Treatment High-Grade Glioma

**DOI:** 10.1155/2021/9437090

**Published:** 2021-05-05

**Authors:** Tao Yuan, Xiaoli Ji, Yawu Liu, Guodong Gao, Jia-Liang Ren, Deyou Huang, Guanmin Quan

**Affiliations:** ^1^Department of Medical Imaging, The Second Hospital of Hebei Medical University, Shijiazhuang, China; ^2^Department of Clinical Radiology, Kuopio University Hospital, Kuopio, Finland; ^3^GE Healthcare China, Beijing, China; ^4^Department of Radiology, Affiliated Hospital of Youjiang Medical University for Nationalities, Baise, China

## Abstract

The imaging signs which can accurately predict survival prognosis after standard treatment of high-grade glioma (HGG) are highly desirable. This study aims to explore the role of new enhancement beyond radiation field (NERF) in the survival prediction in patients with post-treatment HGG. The present study included 142 pathologically confirmed HGG patients who had received standard treatment. NERF, as well as other conventional MR findings and clinical variables, were included in univariate and multivariate analyses for evaluating their impactions on progression-free survival (PFS) and overall survival (OS). Univariate analysis showed that histological grade (*p*=0.008) and NERF (*p*=0.001) were the prognostic variables for poor PFS, whereas histological grade (*p*=0.017), NERF (*p*=0.001), and new subventricular zone enhancement (nSVZE) (*p*=0.001) were prognostic variables for poor OS. The multivariate analysis showed that NERF (HR 3.93; 95% CI 1.93–8.01; *p*=0.001) and nSVZE (HR 3.92; 95% CI 1.95–7.89; *p*=0.001) were the prognostic variables for poor OS. However, only nSVZE was (HR 3.29; 95% CI 2.04–5.28; *p*=0.001) the prognostic variable for poor PFS. When combining the NERF with the clinical and other MR variables, the highest AUC (0.924) and specificity (0.899) for predicting poor OS were achieved. The location of new developed enhancements relevant to high dose radiation field appears to be the main determinant of their prognostic value. Our results suggest that the new enhancement beyond radiation field can improve the survival prediction in patients with HGG after standard treatment.

## 1. Introduction

High-grade gliomas (HGGs) remain a challenging disease in neuro-oncology. HGGs are the most common malignant and rapid-growing tumors [1]. In spite of standard treatment, including gross-total resection, concurrent radiation therapy, and chemotherapy, the overall 5-year survival remains relatively low [2–4]. Therefore, the method, especially the noninvasive imaging techniques, which can accurately predict disease progression after standard treatment is highly desirable so that the salvage therapy, such as reradiation, could be triggered and the patients' progression-free interval time may consequently be prolonged [5].

At present, conventional magnetic resonance (MR) imaging, including T2-weighted imaging (T2WI), fluid-attenuated inversion recovery sequence (FLAIR), and contrast-enhanced T1-weighted imaging (CE-T1WI), is still the preferred imaging method for evaluating glioma patients after treatment [6, 7]. In the response assessment in neuro-oncology (RANO) criteria, CE-T1WI is the most important sequence in evaluation of response and tumor progression in the HGG patients [8]. After standard treatment, the newly appeared enhancement and its size are important factors in estimating the recurrence and prognosis, but the location of the new enhancement is less studied [9]. The new enhancement, which can situate within or beyond the radiation field, is usually caused by tissue response to the surgical trauma, necrosis, and, importantly, tumor recurrence and radiation damage. The new enhancing lesions, especially those located beyond radiation filed, would be the sign of tumor progression. Therefore, these new enhancing lesions probably influence the survival outcome of the patients with post-treatment HGG.

As the new enhancement beyond radiation field (NERF) may be caused by tumor recurrence, we deduced that the new enhancing lesions could influence the survival outcome. Therefore, the purpose of this retrospective study was to explore the value of NERF by combining it with other MR features and clinical variables in survival prediction in the patients with post-treatment HGG.

## 2. Materials and Methods

### 2.1. Patients

We identified consecutive 167 brain HGG patients who had received standard treatment according to National Comprehensive Cancer Network (NCCN) guideline [9] between October 2016 and October 2018. All these patients received tumor gross-total resection, radiation therapy, and chemotherapy (CCRT) after surgery and six cycles of adjuvant temozolomide (TMZ).

The inclusion criteria included the following: (1) the diagnosis confirmed by histopathologic examination; (2) gross-total resection of HGG; (3) with age older than 18 years; (4) having undergone serial MR scans: after resection and before and after CCRT. The patients had successfully undergone at least 3 times post-CCRT MR scans; (5) with follow-up for more than 12 months. The treatment response was defined based on the RANO criteria [7]. We collected the following data, including patients' demographics, pathologic diagnosis, treatment schedule, MR imaging data, and clinical outcomes. The median follow-up span was 24 months (range 21–28). Due to the retrospective nature, the written informed consent was waived. According to RANO criteria, disease progression is defined as presence of newly enhancing lesions of enlargement of preexisting enhancing lesions on CE-T1WI MR series in addition to T2/FLAIR imaging [4]. Progression-free survival (PFS) was defined as the date of surgery to the date of progression or the date of the last follow-up without progression [10]. Overall survival (OS) was defined from the date of surgery to the date of the last follow-up or death [11].

### 2.2. Imaging Data

All subjects underwent 3.0T MR scan (Philips MRI Systems Achieva, Netherlands). The imaging protocol included pre- and postcontrast CE-T1WI, and T2WI)/FLAIR. The CE-T1WI was performed after Gd-DTPA (0.1 mmol per kilogram of body weight of gadobutrol, Gadovist, Bayer Schering Pharma, Berlin, Germany) which was injected at a rate of 3 ml/sec, followed by a 20 ml 0.9% saline flush using the same flow rate. 58 patients underwent dynamic susceptibility contrast (DSC) perfusion MRI with perfusion-weighted gradient-echo echo-planar sequence. Relative cerebral blood volume (rCBV) of new enhancing lesions was calculated when compared to that of the mirror cerebral region.

### 2.3. Imaging Analysis

We reviewed all the follow-up MR data in Picture Archiving and Communication System (PACS). The new enhancing lesions included (1) NERF, which located beyond radiation field, and (2) new subventricular zone enhancement (nSVZE). The enhancement patterns of residual cavity wall included (1) thin-linear enhancement (partial or entire wall enhancement with thickness < 3 mm), (2) thick-linear enhancement (partial or entire wall enhancement of 3–5 mm in thickness), and (3) nodular wall enhancement (with nodular enhancement of 5–10 mm in thickness) [12]. Since thin-linear enhancement might be reactive enhancement and thick-linear/nodular enhancement might be tumor remnant and the microscopical infiltration of tumor cells [11, 12], we categorized the residual cavity wall enhancement into two groups: no enhancement and thin-linear enhancement and thick-linear/nodular enhancement. The MR imaging findings were identified independently by two neuroradiologists (with 5- and 20-year experiences in diagnostic radiology). When a disagreement existed, a consent was reached after consulting another neuroradiologist (with 25-year experiences in diagnostic radiology).

### 2.4. Statistical Analysis

All statistical analyses were performed with SPSS 21.0 for Windows (SPSS Inc., Chicago, IL, USA). The quantitative data were described as mean and standard deviation when they were in normal distribution and as median and quartile, when they were in nonnormal distribution. Categorical variables were analyzed with log-rank test. Cox's proportional hazards model was used for multivariate analysis. Receiver operating characteristic (ROC) curve analysis was employed in determining threshold value, accuracy, sensitivity, and specificity in differentiating poor and good outcomes. A *p* value < 0.05 was regarded as statistically significant. The interobserver consistency between the two neuroradiologists was evaluated with intraclass correlation coefficient (ICC). The survival times were estimated with the Kaplan–Meier methods.

## 3. Results

### 3.1. Baseline Characteristics

Twenty-five of 167 patients were excluded for the followed reasons: (1) only biopsy was made (*n* = 3); (2) with age < 18 years (*n* = 3); (3) lost to follow-up during the first 12 months after gross-total resection (*n* = 9); (4) poor quality of MR images (*n* = 10). Thus, 142 patients were enrolled in this study, with a mean age of 47.4 ± 14.50 years (range 18–73 years). The subjects included 79 male and 63 female patients. The pathological analysis showed that the HGGs were WHO grade III (*n* = 78) and grade IV (*n* = 64).

**Figure 1:**
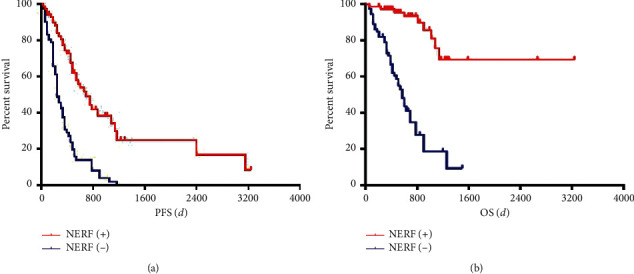
Kaplan–Meier survival curves for PFS (a) and OS (b) according to NERF. NERF, new enhancement beyond the radiation field; PFS, progression-free survival; OS, overall survival.

All patients completed the standard treatment according to the NCCN guideline. The demographics, radiation dose, pathologic characteristics, and imaging features on conventional MRI, as well as clinical outcome are listed in Table 1. The median PFS was 11.50 months (95% confidence interval (CI) 12.73–17.93 months) and median OS was 18.00 months (95% CI 18.70–24.00 months) (Figures 1(a) and 1(b)). 52 patients (36.62%) were dead during the follow-up period.

10 of 23 patients in this series underwent MGMT testing which showed positive MGMT methylation, including 7 patients with NERF lesion and 3 patients without NERF lesions (*p*=0.363). 64 patients underwent IDH genetic testing. But we had not found significant difference between patients with NERF lesion and patients without NERF lesions (*p*=0.130).

In the follow-up MR images, NERF was found in 72 patients, nSVZE in 77 patients, thick-linear and nodular wall enhancement in 78 patients, and nonenhancement hyperintensity on T2WI/FLAIR images in 64 patients. Higher rCBV (1.27; 95% CI 0.538–1.64) was found in 47 of 58 patients who underwent DSC-PWI, including 28 patients with shorter OS and 19 with longer OS (*p*=0.063).

142 HGG patients included the following histological types: 43 patients with anaplastic oligodendroglioma, 30 with anaplastic astrocytoma, 5 with anaplastic pleomorphic xanthoastrocytoma, and 64 with glioblastoma. In terms of new enhancing lesions, there was no significant difference about the incidence of new enhancement beyond radiation field (NERF) between high-grade astrocytoma (56.25%) and high-grade oligodendroglioma (64.28%) patients (*p*=0.492).

### 3.2. Univariate and Multivariate Analyses

Univariate analysis showed that histological grade (*p*=0.008) and NERF (*p*=0.001) were the prognostic variables for poor PFS, whereas histological grade (*p*=0.017), NERF (*p*=0.001), and nSVZE (*p* < 0.001) were prognostic variables for poor OS. The incidence of nSVZE in patients with shorter OS (68.18%, 60/88) was higher than that in patients with longer OS (31.48%, 17/54) (*p* < 0.001). The multivariate analysis (Table 2), NERF (HR 3.93; 95% CI 1.93–8.01; *p* < 0.001), and nSVZE (HR 3.92; 95% CI 1.95–7.89; *p* < 0.001) were confirmed as the prognostic variables for poor OS. However, only nSVZE was (HR 3.29; 95% CI 2.04–5.28; *p* < 0.001) the prognostic variable for poor PFS.

### 3.3. Diagnostic Efficiency of Survival State

The agreement was excellent between the two neuroradiologists for evaluation of nonenhancing T2/FLAIR lesions (ICC 0.931; 95% CI 0.904–0.951; *p* < 0.001), enhancement pattern of residual cavity wall (ICC 0.932; 95% CI 0.905–0.951; *p* < 0.001), NERF (ICC 0.949; 95% CI 0.930–0.964; *p* < 0.001), and nSVZE (ICC 0.951; 95% CI 0.931–0.965; *p* < 0.001).

**Figure 2:**
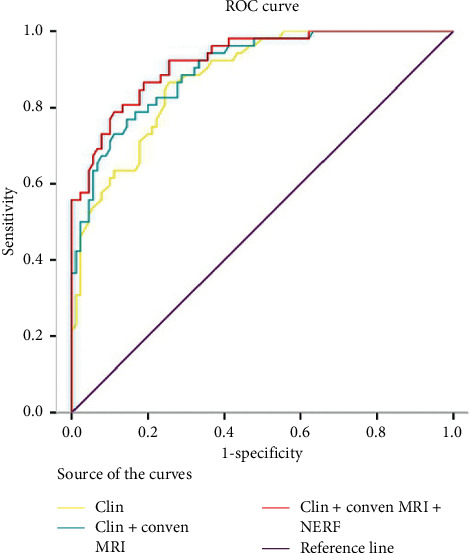
Comparison of ROC curve analyses for different combinations of clinical and MR variables for predicting poor OS. The combination of Clin + Conven MRI + NERF presents the high diagnostic performance (AUC 0.924; 95% CI 0.881, 0.967), with significant level of *p* < 0.001 to any other combinations. ROC, receiver operating characteristic; AUC, area under ROC curve; Clin = age + histological grade; Conven MRI = nonenhanced T2/FLAIR hyperintensity lesions + enhancement of residual cavity wall; NERF, new enhancement beyond the radiation field.

The ROC analysis showed that combination of clinical factors, including age and histologic grade, achieved AUC 0.879 for predicting poor OS with a sensitivity of 0.865, specificity 0.744, and Youden index 0.6108. The diagnostic performance for poor OS was improved when adding conventional MRI factors, including nonenhanced T2/FLAIR hyperintensity lesions and enhancement of residual cavity wall, with AUC of 0.901, sensitivity 0.788, specificity 0.833, and Youden index 0.625. Interestingly, when we added NERF to combination model, which including clinical and conventional MRI factors, the AUC was 0.924, with a sensitivity of 0.788, specificity of 0.889, and Youden index of 0.677 in predicting poor OS (Table 3, Figure 2).

**Figure 3:**
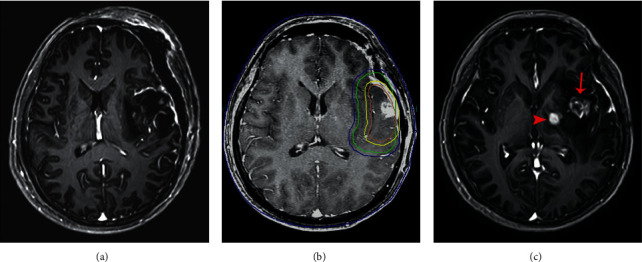
Example of NERF. A 25-year-old female with pathologically confirmed glioblastoma in the left temporal lobe. Her OS was 550 days. Axial contrast-enhanced T1-weighted image 48 h after resection (a). Axial contrast-enhanced T1-weighted image with treatment-planning overlay shows the volumes treated with 60 Gy (yellow outline) and 50 Gy (green outline) (b). The enhancing lesion (arrow) within radiation field and a NERF lesion (arrowhead) outside radiation field in follow-up MRI were detected 24 months after completion of radiotherapy (c). NERF, new enhancement beyond the radiation field.

**Figure 4:**
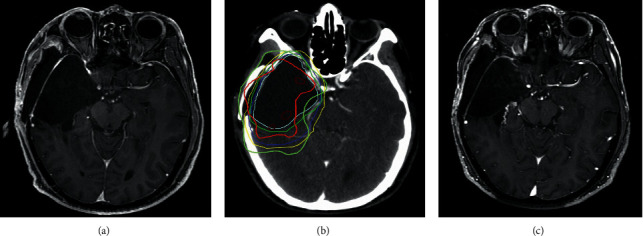
Example of patient without NERF. A 63-year-old male with pathologically confirmed anaplastic astrocytoma in the right temporal lobe. His OS was 790 days. Axial contrast-enhanced T1-weighted image 72 h after resection (a). Axial contrast-enhanced CT image with treatment-planning overlay shows the volumes treated with 60 Gy (red outline) and 50 Gy (yellow outline) (b). There was no new developed enhancing lesion within and outside radiation field in follow-up MRI 6 months after completion of radiotherapy (c). NERF, new enhancement beyond the radiation field.

**Figure 5:**
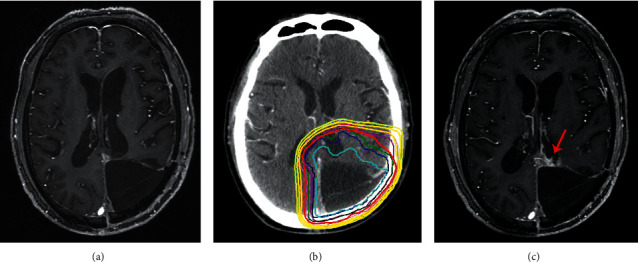
Example of nSVZE. A 66-year-old male with pathologically confirmed anaplastic astrocytoma in the left parieto-occipital lobe. His OS was 510 days. Axial contrast-enhanced T1-weighted image 72 h after resection (a). Axial contrast-enhanced CT image with treatment-planning overlay shows the volumes treated with 60 Gy (blue outline) and 50 Gy (red outline) (b). There was nSVZE lesion at the left trigonum in follow-up MRI 6 months after completion of radiotherapy (c). nSVZE, new subventricular zone enhancement.

Figures 3–5 show the classic examples of patients with NERF, without new enhancing lesion, and with nSVZE.

## 4. Discussion

Predicting the outcome of post-treatment HGG is critical for individualized management. Our study is to evaluate the effect of the location of new enhancement relative to radiation field in discriminating favorable and unfavorable survival prognosis in HGG patients after standard treatment. Our results showed that the new enhancement beyond radiation field, namely, NERF, can be used to improve the accuracy in predicting outcome in those postoperative HGG patients, particularly, when NERF was added to the predicting model.

CE-T1WI is routinely used and is the most important MR sequence in the evaluation of post-treatment HGG patients. In clinical practice, only the increase of enhancement lesions size is considered as an imaging marker of tumor progression according to RANO criteria [4, 8]. The new enhancement could appear in the residual cavity wall and resection area, as well as in the regions which are outside the radiation field. The causes of these new enhancing lesions include both progressive disease and treatment necrosis [13, 14]. We categorized the new enhancement into NERF and nSVZE. The definition of new enhancement in the subventricular zone had been used in recent years in other literatures [5]. However, as far as we know, it is first time to offer the definition of NERF. There are several possible explanations for the NERF, including residual tumor, tumor recurrence, and treatment necrosis. In this study, all the patients underwent gross-total resection of HGG. In the follow-up MR scan at one month after operation, the normal tissue response to the surgical trauma could present as thin-linear enhancement on the residual cavity wall. On the other hand, the residual tumor may lead to thick-linear or nodular enhancement shortly after operation. Thus, CE-T1WI may help for detecting the lesions originated from residual tumor lesions. Moreover, the NERF lesions may come from those so-called tumor stem cells, which can migrate over long distance from the subventricular region and lead to new enhanced lesions [15]. In this series, NERF lesions were found in 58 of 88 patients (65.91%) with shorter OS. In contrast, there was a lower incidence (25.93%, 14/54) of NERF lesions for those patients with longer OS (*p* < 0.001). We may estimate that tumor recurrence which originated from microresidual tumor cells would be the most common cause of NERF lesions and lead to unfavorable outcome.

Our data also showed that nSVZE was a risk factor for a poor outcome. It is interesting that both univariate and multivariate analyses indicated the nSVZE, contact to subventricular zone, which was a strong prognostic variable for both PFS and OS. In our study, 44 of 77 patients with nSVZE had unfavorable survival outcome. Previous studies have shown there is a correlation between preoperative invasion at the subventricular zone and the unfavorable outcome of grade IV gliomas, suggesting an unfavorable impaction of invasion at the subventricular zone on PFS and OS [16–18]. The subventricular zone, which is believed as a pool of stem cells and is within 5 mm to the ventricular wall, may be a structure that the brain tumor stem cells would be derived from [5]. Several studies have shown that aggressive gliomas may be related to the recurrence of neuronal stem-like cells in the subventricular zone [5, 17–19]. When we reviewed the preoperational MRI data, we did not find enhancing lesion contacted to ventricular wall. Thus, we speculated that the nSVZEs in our study were related to the recruitment of those so-called neuronal stem cells. These cells were infinitive to proliferate and could be resistant to standard chemo- and radiotherapy [15]. The salvage radiotherapy with high dose per fraction might even stimulate glioma cell migration [5]. The resistance to the standard and salvage therapies may be the cause of unfavorable PFS and OS in the patients with nSVZE. However, on the other hand, the subventricular zone (SVZ) is significantly more susceptible to radiation damage and this has been well reported. This radiation damage in SVZ includes demyelination, disruption of microvascular structure, and secondary local inflammation. The alteration of SVZ microenvironment would evoke new enhancement in CE-T1WI. Thus, if the new enhancing lesion in SVZ is encountered, its location should be noted relevant to radiation field instead of SVZ.

There are several limitations in this study. First, as a retrospective analysis, only patients with grade III and IV gliomas were enrolled, thus may have a selection bias. In the future, we should include grade II gliomas with wild-type isocitrate dehydrogenase (IDH), which are associated with increased risk of aggressive disease; these grade II gliomas can be defined as so-called “molecular glioblastoma” [20]. The Consortium to Inform Molecular and Practical Approaches to CNS Tumor Taxonomy working committee suggested that histologic grade II and III IDH-wild-type astrocytic glioma should be referred as diffuse astrocytic glioma, IDH-wild-type, for these gliomas containing high-level EGFR amplification or TERT promoter mutations [21]. Second, molecular pathology is an important factor in predicting outcome, including mutation of IDH, O6-methylguanine-DNA methyltransferase (MGMT), and 1p19q co-deletion. The molecular pathology has not been widely included in routine clinical examination, especially in the era before 2016. Since only 23 and 64 patients underwent MGMT and IDH genetic testing separately and no significant difference was found between patients with NERF and those without NERF in our series, we did not analyze the role of biology of HGG in survival prediction. Further analysis should be made for new enhancing lesions based on genetics of HGG in the future. Finally, several other prognostic factors, including relative cerebral blood volume, diffusion characteristics, enhancement lesion size, and salvage therapy, were not included. Although PWI is valuable for differentiation true progression from false progression in post-treatment HGG patients, we had not found significant difference of relative cerebral blood volume (rCBV) between patients with shorter OS and patients with longer OS. Thus, PWI factors were not enrolled in multivariate analysis of survival state.

In conclusion, our data showed that the new enhancing lesion is a useful imaging marker in survival prediction in the HGG patients after standard treatment. The patients with NERF on CE-T1WI after standard therapy may need aggressive and salvage treatments. As CE-T1WI is a convenient, noninvasive, and useful method for evaluating HGG after treatment and the new enhancement can be easily evaluated, further longitudinal studies are needed to confirm our findings, especially the impact of the NERF lesions on survival outcome in the recurrent HGG patients who receive salvage therapy.

## Figures and Tables

**Table 1:** Baseline characteristics of HGG patients (*n* = 142).

Characteristics	Mean/median (standard deviation/95% CI), *n* (%)
Age (years)	47.47 (14.35)
Radiation dose (Gy)	58.92 (5.91)
PFS (months)	345 (382–538)
OS (months)	540 (561–720)

*Gender*	
** **Female	63 (44.37%)
** **Male	79 (55.63%)

*IDH (N* *=* *64)*	
** **Wild	45 (69.35%)
** **Mutation	19 (30.65%)

*Histopathology*	
** **Anaplastic oligodendroglioma	43 (30.28%)
** **Anaplastic astrocytoma	30 (21.13%)
** **Anaplastic pleomorphic xanthoastrocytoma	5 (3.52%)
** **Glioblastoma	64 (45.07%)

*Nonenhanced T2/FLAIR hyperintensity lesions*	
** **Stable or decrease	80 (54.93%)
** **Increase	62 (45.07%)

*Enhancement of residual cavity wall*	
** **Nonenhancement or thin-linear enhancement	64 (45.07%)
** **Thick-linear or nodular enhancement	78 (54.93%)

*PWI* (*N* *=* *58*)	
** **Progress	47 (81.03%)
** **Stable	11 (18.97%)

*NERF*	
** **Negative	70 (49.30%)
** **Positive	72 (50.70%)

*nSVZE*	
** **Negative	65 (45.77%)
** **Positive	77 (54.26%)

*Outcome*	
** **Remain alive till due time	90 (63.38%)
** **Dead	52 (36.62%)

HGG, high-grade glioma; CI, confidence interval; PFS, progression-free survival; OS, overall survival; IDH, isocitrate dehydrogenase; FLAIR, fluid-attenuated inversion recovery; NERF, new enhancement beyond the radiation field; nSVZE, new subventricular zone enhancement.

**Table 2:** Multivariate analysis of prognostic based on OS.

Characteristics	Favorable prognosis			Poor prognosis		
	*β*	OR (95%CI)	*p* value	*β*	OR (95%CI)	*p* value
Histological grade	1.262	3.533 (0.217, 46.143)	0.336	−0.923	0.397 (0.132, 1.198)	0.101
NERF	−2.535	0.079 (0.006, 1.052)	0.055	−1.802	0.165 (0.0.045, 0.605)	0.007
nSVZE	−0.07	0.468 (0.094, 2.328)	0.353	−0.541	0.582 (0.167, 2.026)	0.395

OS, overall survival; favorable prognosis, OS ≥ 660 days; poor prognosis, OS＜660 days; *β*, regression coefficient; OR, odds ratio; FLAIR, fluid-attenuated inversion recovery; NERF, new enhancement beyond the radiation field; nSVZE, new subventricular zone enhancement.

**Table 3:** ROC curve analyses of diagnostic performance of combinations of various variables.

Characteristics	AUC	95% CI	Sensitivity	Specificity	Youden index	*p* value
Clin	0.879	(0.825, 0.933)	0.865	0.744	0.610	＜0.001
Clin + Conven MRI	0.901	(0.851, 0.950)	0.788	0.833	0.625	＜0.001
Clin + Conven MRI + NERF	0.924	(0.881, 0.967)	0.788	0.889	0.677	＜0.001

ROC, receiver operating characteristic; AUC, area under ROC curve; Clin = age + histological grade; Conven MRI = nonenhanced T2/FLAIR hyperintensity lesions + enhancement of residual cavity wall; NERF, new enhancement beyond the radiation field.

## Data Availability

The data used to support the findings of this study are available from the corresponding author upon request.
